# Development of plastic-degrading microbial consortia by induced selection in microcosms

**DOI:** 10.3389/fmicb.2023.1143769

**Published:** 2023-04-11

**Authors:** Jesús Salinas, Víctor Carpena, María R. Martínez-Gallardo, Martín Segado, María J. Estrella-González, Ana J. Toribio, Macarena M. Jurado, Juan A. López-González, Francisca Suárez-Estrella, María J. López

**Affiliations:** Unit of Microbiology, Department of Biology and Geology, CITE II-B, Agrifood Campus of International Excellence ceiA3, CIAIMBITAL, University of Almeria, Almeria, Spain

**Keywords:** biodegradation, LLDPE, microbial consortium, microcosm, microplastic, plastic film, petroleum-based plastics, sequential enrichment

## Abstract

The increase in the production of highly recalcitrant plastic materials, and their accumulation in ecosystems, generates the need to investigate new sustainable strategies to reduce this type of pollution. Based on recent works, the use of microbial consortia could contribute to improving plastic biodegradation performance. This work deals with the selection and characterization of plastic-degrading microbial consortia using a sequential and induced enrichment technique from artificially contaminated microcosms. The microcosm consisted of a soil sample in which LLDPE (linear low-density polyethylene) was buried. Consortia were obtained from the initial sample by sequential enrichment in a culture medium with LLDPE-type plastic material (in film or powder format) as the sole carbon source. Enrichment cultures were incubated for 105 days with monthly transfer to fresh medium. The abundance and diversity of total bacteria and fungi were monitored. Like LLDPE, lignin is a very complex polymer, so its biodegradation is closely linked to that of some recalcitrant plastics. For this reason, counting of ligninolytic microorganisms from the different enrichments was also performed. Additionally, the consortium members were isolated, molecularly identified and enzymatically characterized. The results revealed a loss of microbial diversity at each culture transfer at the end of the induced selection process. The consortium selected from selective enrichment in cultures with LLDPE in powder form was more effective compared to the consortium selected in cultures with LLDPE in film form, resulting in a reduction of microplastic weight between 2.5 and 5.5%. Some members of the consortia showed a wide range of enzymatic activities related to the degradation of recalcitrant plastic polymers, with *Pseudomonas aeruginosa* REBP5 or *Pseudomonas alloputida* REBP7 strains standing out. The strains identified as *Castellaniella denitrificans* REBF6 and *Debaryomyces hansenii* RELF8 were also considered relevant members of the consortia although they showed more discrete enzymatic profiles. Other consortium members could collaborate in the prior degradation of additives accompanying the LLDPE polymer, facilitating the subsequent access of other real degraders of the plastic structure. Although preliminary, the microbial consortia selected in this work contribute to the current knowledge of the degradation of recalcitrant plastics of anthropogenic origin accumulated in natural environments.

## 1. Introduction

Plastics are synthetic polymers derived from petroleum that have displaced natural products due to their intrinsic properties, such as durability and versatility. These properties make them ideal materials for use in a variety of industry sectors, mainly focused on food and agriculture (Sivan, 2011; Rochman et al., 2019). As a consequence, its use has increased exponentially reaching 370 million tons per year in Europe in 2016 and forecasting 800 million tons per year by 2040 (Lebreton and Rady, 2019; Wang et al., 2019). This high production results in the generation of large quantities of plastic waste that is highly resistant to biodegradation, mainly due to its molecular structure, which is made up of long carbon chains (Kučić Grgić et al., 2021; Ragusa et al., 2021). Pollution by microplastics and film plastics is increasingly problematic due to their small size, which allows their easy ingestion by a wide range of organisms and their consequent accumulation in food chains (Zurier and Goddard, 2021). At the ecosystem level, pollution by these plastics causes numerous negative effects. Specifically, they can cause physical harm to both marine and terrestrial animals, particularly if they become entangled in plastic debris (Zettler et al., 2013). Additionally, microplastics have the potential to absorb toxic chemicals and pollutants from the environment, which can be transferred to organisms that consume them, leading to various health issues and even death (Jabeen et al., 2017). It is also worth noting that plastic waste can significantly alter habitats and disrupt natural ecosystems, ultimately leading to changes in the populations and distribution of species (Evode et al., 2021). These factors highlight the urgent need to address the current problem of plastic pollution and it is far-reaching environmental, ecosystem and health consequences. In addition, the scarcity of effective methods for recycling plastics means that this type of waste accumulates in large quantities in the environment, an emerging concern for human health and natural ecosystems (Alshehrei, 2017).

The heterogeneous and multilaminated composition of most plastics is responsible for the fact that most recycling processes are considered to be of low efficiency in the absence of mechano-chemical treatments that allow optimal reuse (Gopinath et al., 2020). Therefore, the need arises to look for biological tools that allow the efficient degradation and/or transformation of plastic waste, thus eliminating its harmful accumulation in the environment. In this sense, several authors have postulated the biodegradation of plastics by microorganisms and their enzymatic mechanisms as one of the most suitable and sustainable alternatives for the bioremediation of plastic waste (Karlsson et al., 2018). According to recent studies, some enzymes such as lipases or cutinases have been described as potential candidates for the biodegradation of plastic because the substrates to which they specifically bind show some structural similarity to some plastic materials. The degradation of plastics in nature is influenced by both microbial activity and abiotic factors, which play a crucial role in the process. Specifically, abiotic factors such as light, heat, humidity, and pH have been shown to facilitate the fragmentation of plastics by breaking the bonds present in the polymer structure and promoting the formation of new functional groups, thus altering their structural homogeneity (Siracusa, 2019). Exposure to water, UV radiation, or high temperatures can trigger chemical reactions that lead to molecular destabilization and initiate the degradation process. Catalysis, thermal, photo-oxidative, or ozone degradation can reduce the plastic crystallinity (Manzoor et al., 2022), which is a key factor in its susceptibility to degradation and the emergence of microplastics (<5 mm) and nanoplastics (<0.1 μm). The intricate interplay between microbial activity and abiotic factors in plastic degradation underscores the complexity of the process and the need for a comprehensive understanding of the underlying mechanisms. The degradation of polyethylene, one of the most widely used plastics in industry and agriculture, is a challenge from a biotechnological and enzymatic point of view due to some of its intrinsic characteristics such as high molecular weight, hydrophobicity and absence of functional groups attackable by enzymatic mechanisms (Nowak et al., 2011; Hosaka et al., 2013; Restrepo-Flórez et al., 2014). Considering the high recalcitrance of this type of plastic, the search for enzymes for its biodegradation focuses attention on ligninolytic enzymes such as laccases, ligninases and manganese peroxidases, due to their ability to degrade structurally complex compounds such as lignin and its derivatives (Molitor et al., 2020; Temporiti et al., 2022).

On the other hand, axenic microbial cultures commonly used for the biodegradation of plastic have had limited success, which encourages the improvement of this strategy by using microbial consortia that act synergistically as an optimized alternative (Peixoto et al., 2017). Consortia are more stable than pure cultures, as they can adapt more easily to different ecosystems, tolerate environmental changes better (Bhatia et al., 2018), and exhibit greater metabolic versatility. To obtain a microbial consortium capable of biodegrading plastic, it is hypothesized that the ideal environment for the selective search of these microorganisms is a habitat contaminated with plastic, since the microorganisms present are able to tolerate this pollutant and even use it metabolically (Ru et al., 2020). Taking into account the aforementioned background, we hypothesize that the selection of natural consortia obtained from soil microcosms amended with the target plastics (LLDPE in this case) would ensure to get the combination of microbial components with complementary metabolism for the degradation of the target plastic. Therefore, the novelty of this work lies in obtaining microbial consortia with the potential to biodegrade a scarcely studied plastic such as linear low-density polyethylene (LLDPE) from an artificially contaminated microcosm, thus inducing a selective pressure, and with the proliferation of plastic degrading microorganisms (Henry et al., 2021). While other plastics such as polyethylene terephthalate have been extensively studied because they contain functional groups that facilitate their biodegradation, there is a notable lack of knowledge about the biodegradation of plastic with a more crystalline molecular structure such as polyethylene (Wierckx et al., 2018).

Based on the foregoing, this study aims to select and characterize a stable plastic-degrading microbial consortium by induced and sequential enrichment from plastic-contaminated soil microcosms. In parallel, the efficiency of plastic degradation by consortium members will be verified, as well as their molecular identification and enzymatic profiles related to the degradation of recalcitrant plastics.

## 2. Materials and methods

### 2.1. Establishment of the microcosm

The establishment of a microcosm artificially contaminated with LLDPE was essential to initiate the selective induced enrichment protocol, and to favor the proliferation of a microbial community adapted to this type of contaminant. For this purpose, two 5 × 5 cm^2^ pieces of LLDPE in film format (Jolly Plastic S.P.A., Italy) were buried in a 500 mL capacity container with 300 g of agricultural soil located at the southeast of Spain (Geolocation: 36.826165, −2.438383). Plastic sterilization was carried out by immersion in 70° alcohol for 45 min and subsequently dried at 40°C under sterile conditions. The containers were incubated for 3 months in the dark at 30°C. Humidity was maintained between 40–50% by bimonthly humidification with sterile water.

### 2.2. Induced selective enrichment protocol

**Figure 1:**
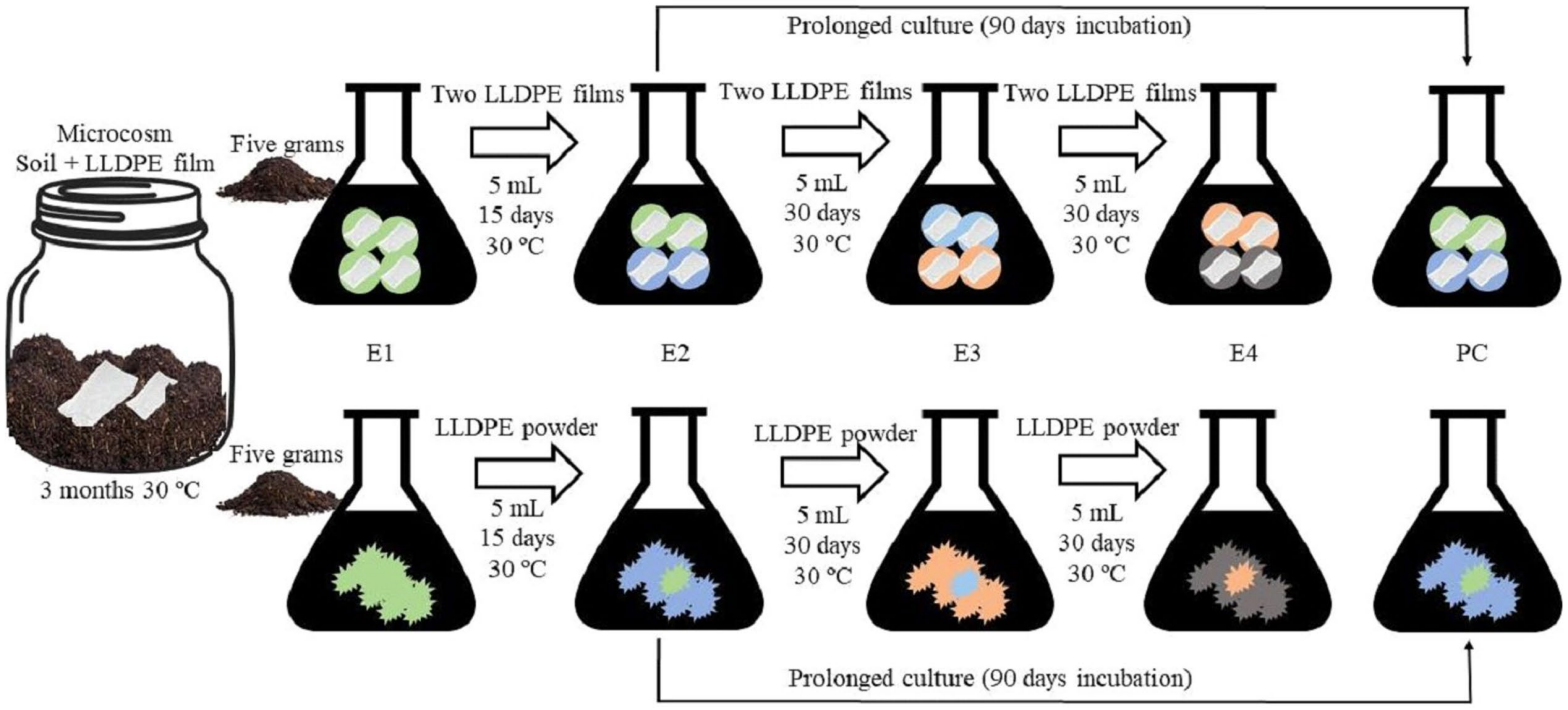
Experimental design. Sequential enrichments with plastic (film/powder) as the sole carbon source from a microcosm artificially contaminated with LLDPE. E1 was based on the transfer of 5 g of soil from the induced microcosm in two flasks with MSM and LLDPE (film or powder). The rest of the transfers included 5 mL of liquid medium and plastic (film or powder) from the previous enrichment culture to a fresh medium supplemented with sterile plastic (film or powder). E1, E2, E3, and E4: Enrichments 1, 2, 3, and 4. PC: Prolonged culture.

To execute the first step of the sequential enrichment protocol (E1), two strategies were carried out in parallel, using LLDPE in powder or film form (Figure 1). The powder LLDPE was obtained through a well-established method that involved grinding plastic pellets in an ultra-centrifugal mill ZM 300 (Retsch) with 50 mL of liquid nitrogen, resulting in a particle size of less than 0.5 mm in diameter. For this purpose, 5 g of the artificially contaminated microcosm, was incorporated with 50 mL of minimal saline medium (MSM) into two 250 mL flasks (Janshekar et al., 1982). Afterward, one of the flasks was enriched with 1% (w/v) LLDPE powder (<500 μm) and the other one with four 1 × 1 cm^2^ films (Li et al., 2020; Liu et al., 2023), as the sole carbon source (Figure 1). Subsequently, periodic transfers to fresh enrichment medium were performed by adding 5 mL of the previous E1 culture, in the case of the LLDPE powder-based assay, and two pieces of LLDPE plastic also from E1, in the case of the film-based assay (Figure 1). MSM with 1% (w/v) LLDPE powder or 2 pieces of fresh LLDPE plastic in the case of the film-based enrichments was used in all enrichment steps. The sequential enrichment operation (Figure 1) was repeated up to 4 times (E1-E4). Incubation conditions in all enrichment phases were 30°C and constant agitation at 110 rpm for 30 days for each phase, except for E1, which was incubated for 15 days.

### 2.3. Counts and characterization of plastic degrading microorganisms

The study of the microbial community present in each enrichment culture was carried out in order to promote the selective development of those microorganisms with the capacity to degrade the plastic. For this purpose, samples were taken from the two constituent flasks of each sequential phase. The presence of total bacteria, as well as total fungi (molds and yeasts), were quantified by inoculation on Petri plates with nutrient agar (NA) and rose bengal agar (RB), respectively. Taking into account the relationship between the degradation of plastics and other recalcitrant materials such as lignin, it was considered of special interest to carry out the quantification of ligninolytic microorganisms in Remazol Brilliant Blue R (RBBR) medium (Kirk et al., 1986; Martínez-Gallardo et al., 2020). Reagents were provided by the company Panreac (Barcelona, Spain). In each enrichment phase, the majority of morphotypes of bacteria and fungi were isolated from the Petri dish counts. In the case of the RBBR medium, those positive morphotypes associated with ligninolytic activity were isolated, after verifying that they had not been selected in previous phases.

It should be noted that most plastics contain additives that are easily degradable by microorganisms and, therefore, more accessible as an immediate carbon source. Therefore, based on this assumption, these additives could be degraded by microorganisms in less time than the plastic polymer. For this reason, once all the sequential enrichments had been completed, a microbial count was performed on the samples from stage E2, after they had been incubated for 90 days. This new sampling (Figure 1) was referred to as prolonged culture (PC). Additionally, the microorganisms isolated by sequential enrichment from the microcosms artificially contaminated were characterized based on their enzymatic activities associated with plastic degradation. Thus, laccase (LAC1 and LAC2), polyphenol oxidase (PO), polyurethanase (PU), cutinase (CUT1 and CUT2), lipase (LIP) and ligninase (RBBR) activities were qualitatively analyzed according to the methodology previously described (López et al., 2006; Martínez-Gallardo et al., 2020; Molitor et al., 2020).

### 2.4. Gravimetric measurement of the degradation efficiency of LLDPE

At the end of the incubation period (E4), plastic degradation was analyzed gravimetrically. To remove the microbial biomass adhering to the surface of the plastic, the liquid medium was filtered and the microplastic retained on the filter was kept in contact with a 50 mL solution of 2% (w/v) sodium dodecyl sulfate (SDS) for 4 h. After this time, SDS was removed and plastic was washed on the filter paper with abundant distilled water until the plastic was free of residues. The weight loss (%) was calculated using the following formula (Skariyachan et al., 2021): Weight loss (%) = ((Initial weight - Final weight) / Initial weight) × 100.

### 2.5. Evaluation of the impact of biodegradation on the plastic surface by high-resolution field emission scanning electron microscopy (FESEM)

To visualize and evaluate the interaction of microorganisms on the surfaces of LLDPE plastic films, an analysis based on High-Resolution Field Emission Scanning Electron Microscopy was performed. For this purpose, squares of plastic sheets of approximately 4×4 cm were initially cut and sterilized according to the procedure described in section 2.1. The strains with the best growth capacity from the E4 and PC enrichments were evaluated. The films were placed in sterile Petri dishes and 100 μL of a cell suspension (10^5^ CFU/mL) in carbon-free minimal salt medium was added every 2 days for 3 months. Untreated films and films in which non-inoculated MSM was incorporated were used as controls. After the incubation period, the samples were sonicated for 5 min at 40 kHz to remove salt crystals and biofilms generated on the LLDPE pieces. They were then washed with sterile distilled water. The biodegradation impact on the plastic film was evaluated by High-Resolution Field Emission Scanning Electron Microscopy (FESEM).

### 2.6. Identification of LLDPE degrading microorganisms

The identity of the selected isolates was determined by molecular methods. Bacterial identification (including actinobacteria) was determined based on amplification and sequencing of the 16S rRNA gene, while the 5.8S-ITS ribosomal region was used for yeast identification. For the extraction of genomic DNA, two fresh bacterial colonies grown on Nutrient Agar (NA) or 2 yeast-like colonies grown on Rose Bengal Agar were suspended in 500 μL of 0.9% ClNa (w/v) before proceeding with DNA extraction. Subsequently, the suspensions were heat shocked at 97°C for 5 min and finally placed in an ice bath for 5 min. Universal primers 27F (5′-AGAGTTTGATCATGGCTCAG-3′) and 907R (5′-GGTTACCTTGTTACGACTT-3′) were used for bacterial genomic DNA by polymerase chain reaction (Isik et al., 2014). The PCR amplification procedure was carried out under the following conditions: initial denaturation at 95°C for 10 min, followed by 40 cycles of denaturation at 95°C for 1 min, annealing at 62°C for 30 s, and extension at 72°C for 30 s, end up in a single final extension step at 72°C for 10 min. For PCR amplification of fungal DNA, the universal primers ITS1 (50-TCCGTAGGTGAACCTGCGG-30) and ITS4 (5’-TCCTCCGCTTATTGATATGC-3′) were used (Jurado et al., 2014). The PCR amplification protocol employed for fungal DNA involved an initial denaturation step at 95°C for 10 min, followed by 40 cycles of denaturation at 95°C for 30 s, annealing at 57°C for 45 s, and extension at 72°C for 1 min. The amplification program culminated in a single final extension step at 72°C for 10 min. The amplified fragments of DNA were subjected to electrophoresis in 1% agarose stained with GelRed (Biotium Inc., Hayward, CA, United States), followed by visualization and imaging using a gel documentation system. The amplicons obtained from PCR amplification of 5.8S and 16S rDNA were sequenced by Sequencing Service at the University of Almeria (SSA) *via* the dideoxynucleotide cycle sequencing method on an ABI 3500 genetic analyzer (Applied Biosystems, Foster City, CA, United States). The forward and reverse sequences were edited, assembled and aligned using the programs Sequence Scanner v1.0 (Applied Biosystem), Reverse Complement,^1^ Clustal X v2.0.11, and MEGA 5 v5.2. The partial or nearly full-length sequences were compared for similar nucleotide sequences with the BLAST search of the National Center of Biotechnology Information (NCBI, http://blast.ncbi.nlm.nih.gov/Blast.cgi). The similarity among 16S rRNA gene sequences obtained from the bacterial and yeast isolates was assessed by a multiple alignments using ClustalW and phylogenetic analysis by the statistic of maximum likelihood conducted using the software MEGAX v11.0.13 (Kumar et al., 2018).

### 2.7. Statistical analyses

Analysis of variance (ANOVA; *p* < 0.05) was carried out for all variables defined over the experiments. Fisher’s LSD (Least Significant Difference) test to compare the mean values of the different treatments and determine if there were any significant differences between them. The results of the statistical analysis have been presented in the form of figures with error bars representing the LSD interval. The bars bearing different letters were found to be significantly different according to Fisher’s LSD test (*p* < 0.05). All experimental conditions and analyses were performed in triplicate and data are presented as the mean. Formerly, data distribution normality and homogeneity were confirmed by employing the Saphiro-Wilk test. Statistical analyses were carried out using Statgraphics Centurion XIX version 19.4.01 (Stat-Point, Inc.).

## 3. Results

### 3.1. Counting and morphotyping the plastic degrading microorganisms during the induced enrichment protocol: Obtaining the plastic degrading consortium

**Figure 2:**
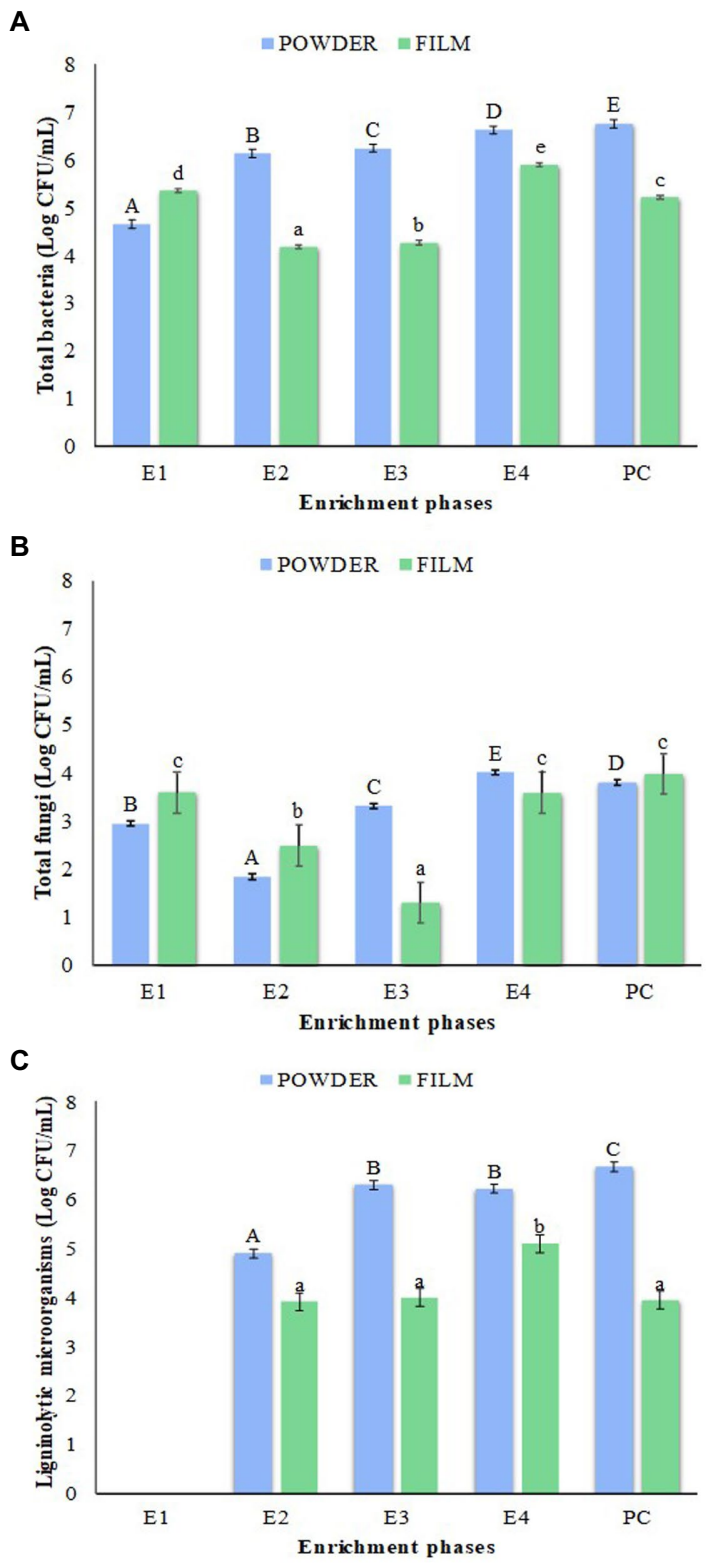
Counts of total bacteria **(A)**, total fungi **(B)**, and ligninolytic microorganisms **(C)** on the enrichment process with LLDPE powder (blue bars) and film (green bars), in addition to PC. E1, E2, E3 and E4: Enrichments 1, 2, 3 and 4; PC: Prolonged culture. The values are the average of three replicates. Error bars represent LSD interval, the bars bearing different letters were significantly different according to Fisher’s LSD test (*p* < 0.05) (uppercase letters for film format of LLDPE and lowercase letters for film format).

Total bacteria, molds and yeasts, as well as ligninolytic microorganisms present in each enrichment culture were quantified (Figure 2). The highest counts of total bacteria and ligninolytic microorganisms were observed in the enrichments obtained with LLDPE in form of ground powder instead of film highlighting the prolonged culture (PC) reaching the maximum population levels 6.66 Log CFU/mL for both microbial groups (Figures 2A,C). It should be noted that the group of ligninolytic microorganisms was undetectable in phase E1 in RBBR medium (Figure 2C). In general, mold and yeast counts were lower but very similar for both types of enrichment (LLDPE powder and film). Although a slight increase in fungal microbiota was detected at the end of the selective enrichment protocol (E4), as well as in the prolonged culture (PC), the levels did not exceed 4.0 Log CFU/mL (Figure 2B).

**Figure 3:**
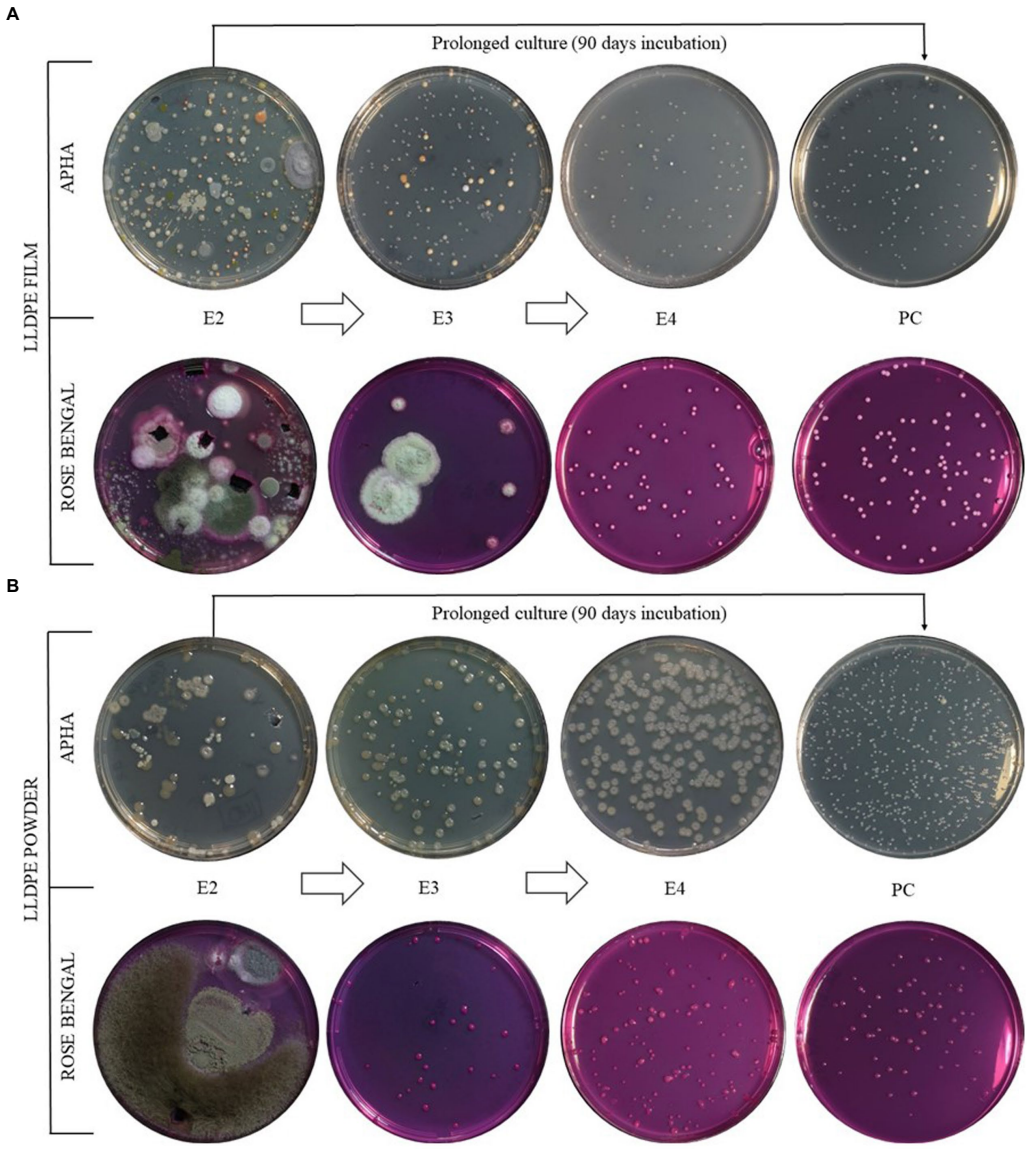
Colonial morphotypes comparison between sequential enrichment with LLDPE film **(A)** and powder **(B)**. From top down, bacterial (NA medium) and fungal (Rose Bengal medium) morphotypes. E1, E2, E3 and E4: Enrichments 1, 2, 3 and 4; PC: Prolonged culture.

The colonial morphotyping during the enrichment process revealed a reduction in microbial diversity in each transfer, resulting in stable microbial consortia in both film and powder LLDPE-containing cultures (Figures 3A,B). In all cases, a decrease in the biodiversity of morphotypes present during the sequential enrichment phases was observed. It is worth noting the disappearance of filamentous fungi after E2 phase in both enrichment formats, powder and film. In particular, enrichment of the samples on powdered LLDPE from E1 to E4 phases revealed the presence of a consortium consisting mainly of two morphotypes: one bacterial and the other yeast-forming. The same scenario was observed in the enrichment from LLDPE in film format, where three bacterial morphotypes and another one yeast-like remained at the end of the assay (E4). In order to clarify whether these microorganisms were feeding on LLDPE plastic and not exclusively on additives, a comparison was made between the different morphotypes present in the prolonged culture (PC), after 3 months of incubation from E2, and E4, with only 30 days of incubation (Figure 3).

This was intended to demonstrate whether the population likely to degrade LLDPE in the prolonged culture (PC) also degraded LLDPE in E4 or, on the contrary, the population in E4 degraded exclusively the additives accompanying the polymer added to the culture. According to the results obtained, the REBF6 and RELF10 morphotypes, observed in PC, were also detected in the E4 enrichments with LLDPE in film form (Table 1). On the other hand, the REBP5 morphotype observed in PC was also detected in the E4 enrichment with LLDPE in powder form. However, REBF2, REBF3 and RELP11 morphotypes were detected exclusively in E4, while REBP7 and RELP9 were detected exclusively after PC (Table 1). The detection of microbial morphotypes in E4 that did not grow in PC could be indicative of their ability to grow only from readily metabolizable additives that accompany LLDPE in E4. However, morphotypes detected on PC but not on E4 could be potentially plastic-degrading microorganisms under the conditions experimented in this work.

**Table 1:** Enzymatic activities.

Strain	Enrichment Phase	Plastic format	Group	Identity	Acc. Number	Enzymatic activities							
						LIP	PU	CUT1	CUT2	LAC1	LAC2	PO	RBBR
REBF1	E4	Film	Bacteria	*Castellaniella denitrificans*	KF371657.1	0	0	1	0	0	0	0	0
REBF2	E4	Film	Bacteria	*Rhodococcus sp.*	MK854905.1	1	0	1	0	0	0	0	1
REBF3	E4	Film	Bacteria	Non identified		1	0	1	0	0	0	0	1
RELF8	E4	Film	Fungi	*Debaryomyces hansenii*	MK394103.1	0	0	0	0	0	0	0	1
REBF6	PC	Film	Bacteria	*Castellaniella denitrificans*	KF371657.1	0	0	0	0	0	0	0	1
RELF10	PC	Film	Fungi	*Debaryomyces hansenii*	MK394103.1	1	0	1	0	0	0	0	1
REBP4	E4	Powder	Bacteria	*Pseudomonas aeruginosa*	MT633047.1	1	0	1	0	0	0	1	1
RELP11	E4	Powder	Fungi	Non identified		1	0	0	0	0	0	0	1
REBP5	PC	Powder	Bacteria	*Pseudomonas aeruginosa*	MT633047.1	1	0	1	0	0	0	1	1
REBP7	PC	Powder	Bacteria	*Pseudomonas alloputida*	CP045551.1	0	0	1	1	0	0	1	1
RELP9	PC	Powder	Fungi	Non identified		1	0	1	1	0	0	0	1

LIP, Lipase; PU, Polyurethanase; CUT1, Cutinase 1; CUT2, Cutinase 2; LAC1, Laccase 1; LAC2, Laccase 2; PO, Polyphenol oxidase; RBBR, Ligninase; 0, negative; 1; positive.

The following bacterial strains were deposited in public database GenBank: *Rhodococcus* sp. strain REBF2 OQ411267; *Castellaniella* sp. strain REBF6 OQ411268; *Pseudomonas aeruginosa* strain REBP4 OQ411269; *Pseudomonas alloputida* strain REBP7 OQ411270; *Debaryomyces hansenii* isolate RELF8 OQ561300. These strains were taxonomically classified using their 16S rRNA gene and ITS region sequences (Supplementary Figure S1).

### 3.2. Gravimetric measurement of the degradation efficiency of LLDPE

Once the enrichment protocol was completed, the weight loss of the powdered LLDPE was studied in the E4 Enrichment phase in order to check the degradation of the plastic by the microorganisms present in the cultures. This study was intentionally performed during this enrichment phase, taking into account that no material was transferred back to a fresh medium from this point onwards. Thus, it is understood that the variation detected in the weight of powder LLDPE corresponds entirely to microbial action and not to the transfer of the culture. After applying the protocol described in section 2.4., a weight loss between 2.5–5.5% could be detected in the powdered LLDPE after 30 days of enrichment in E4.

### 3.3. Characterization and molecular identification of consortium partners

A total of seven bacterial and four fungal strains (yeast-like) corresponding to eight different morphotypes from PC and E4 enrichment were selected for enzymatic characterization and molecular identification (Table 1). The members of the potential plastic degrading consortia were characterized by the expression of previously reported enzymatic activities in association with plastic biodegradation. That is the case of laccase (LAC1 and LAC2), polyphenol oxidase (PO), polyurethanase (PU), cutinase (CUT1 and CUT2), lipase (LIP) and ligninase (RBBR) activities. All strains expressed at least one enzymatic activity related to plastic biodegradation. Some strains were able to express up to four of the tested enzyme activities (*Pseudomonas aeruginosa* REBP4 and REBP5, *P. alloputida* REBP7) and Non identified-Yeast RELP9. Notably, the activities most represented in the consortium members were ligninase (RBBR), and cutinase type 1 (CUT1).

### 3.4. Evaluation of the impact of biodegradation on the plastic surface by FESEM

**Figure 4:**
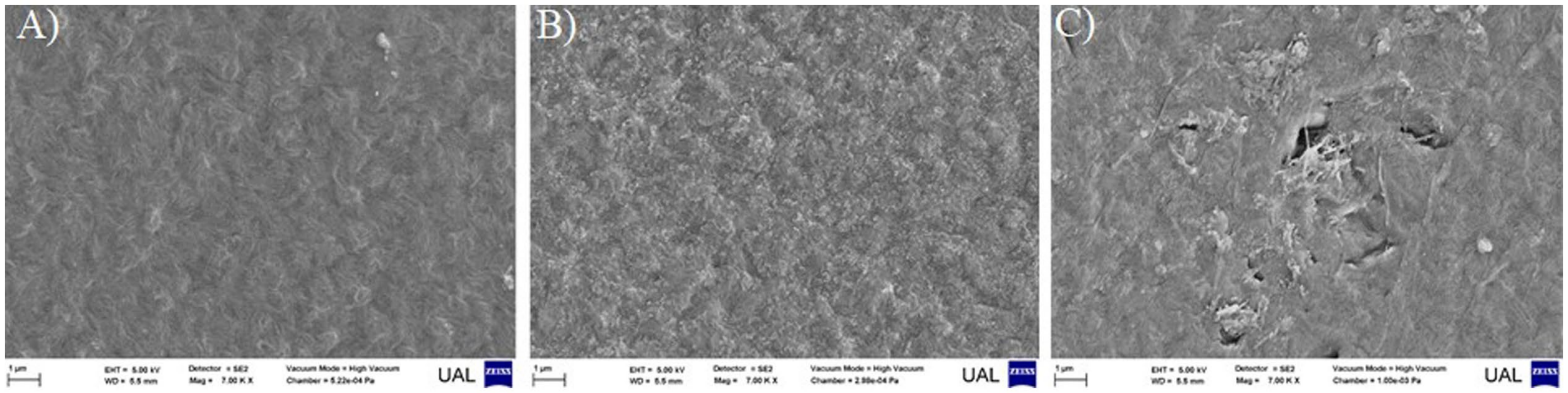
Micrographs of the LLDPE film surface observed by FESEM after 3 months of incubation. **(A)** LLDPE films no treated with MSM medium nor microbial inoculum, **(B)** LLDPE film treated with MSM medium without carbon nor inoculum, and **(C)** LLDPE film inoculated with *P. alloputida* REBP7 in MSM medium without carbon.

Based on the expression of a wide range of enzymatic activities related to plastic degradation (Table 1), the strain *P. alloputida* REBP7 was selected for performing a biodegradation impact analysis on LLDPE film. On the other hand, this morphotype had been isolated exclusively in PC enrichment. For this reason, it was an interesting candidate to be a consortium member plastic-degrading. After 3 months of incubation under the conditions described in section 2.5., deterioration of the LLDPE surface were observed by FESEM in the inoculated samples. (Figure 4C). In this case, the impact of degradation was evident through the observation of craters. On the contrary, anomalies on the plastic surface were not detected in both treated samples with MSM medium and non inoculated samples (Figures 4A,B). This fact supported the biodegradation capacity of the LLDPE by the strain *P. alloputida* REBP7.

## 4. Discussion

Most of the techniques to obtain microorganisms capable of biodegrading plastics are based on the search of habitats naturally contaminated by recalcitrant compounds. In this work, enrichment techniques have been developed from microcosms artificially contaminated with LLDPE in different formats to obtain stable microbial consortia. The microbial load increased with each transfer as well as the reduction of biodiversity, revealing a very likely adaptation of microorganisms to a minimal medium with LLDPE as the sole carbon source (Curiel-Alegre et al., 2022). Similar results were obtained by Liu et al. (2023), who used a sequential enrichment protocol using expanded polystyrene from a mangrove-type ecosystem as inoculum and sole carbon source to obtain polystyrene-degrading microorganisms. These authors observed a decrease in biodiversity with each enrichment, although the protocol allowed the isolation of a high diversity of polystyrene-degrading strains.

When looking for microorganisms with the ability to biodegrade plastic materials, it is reasonable to look for them among those capable of degrading other recalcitrant natural polymers such as lignin. Recent research has demonstrated that ligninolytic microorganisms are enrolled in plastic degradation due to the production of nonspecific oxidative enzymes such as laccase, manganese peroxidase or lignin peroxidase (Daly et al., 2021; Kavitha and Bhuvaneswari, 2021). Therefore, in this work, potential ligninolytic microorganisms isolated from RBBR medium, could be considered as potential degraders of plastic polymers. Such is the case of the bacterial strains *P. alloputida* REBP7 and *P. aeruginosa* REBP5.

Results obtained in this work regarding plastic biodegradation, showed how the format of the plastic can interfere with the level of biodegradation and consequently with the induced strain selection. In this sense, the LLDPE in form of ground powder seems to be more susceptible to biodegradation than the film format. This may be logical considering that the powder format has a larger specific contact surface, which is more susceptible to attack by microorganisms (Pischedda et al., 2019).

Recent studies support that microorganisms have a more prominent capability to degrade plastic additives (such as flame retardants or high molecular weight plasticizers at high concentrations), before than plastic polymers. This could be explained because they are more accessible for microbial enzymes than polymers based on C-C bonds (Carmen, 2021). In this study, it was inferred that the microorganisms present in enrichment 4 (E4) and prolonged culture (PC) were true plastic degraders, while those present exclusively in E4 were additive degrading microorganisms. With this premise, several strains of interest were selected in this study according to their potential to degrade LLDPE in film or powder form, after applying the selection protocol in microcosms. In view of the results obtained, it is evident that the composition of the most effective consortia could be highly variable depending on whether LLDPE in film or powder form is used. In the case of the strains selected from the LLDPE enrichment protocol in film form, two strains were isolated in the E4 enrichment, *Castellaniella denitrificans* REBF1 and *Debaryomyces hansenii* RELF8. Both strains were directly related to LLDPE degradation since the same mophotypes were identified in prolonged cultures (PC), *Castellaniella denitrificans* REBF6 and *Debaryomyces hansenii* RELF10. Consequently, the species *C. denitrificans* and *D. hansenii* are proposed to form an effective consortium for the degradation of LLDPE. Two other strains only present in the E4 enrichments with LLDPE in film format, *Rhodococcus* RBF2 and Non identified-Bacteria REBF3, could be attributed with the ability to degrade the additives that accompany the plastics, which would also serve to facilitate the access of the plastic degraders to the more complex structure of the polymer. Although the genus *Rhodococcus* has previously been described as a voracious devourer of plastic additives (Lumio et al., 2021), little is known about its ability to degrade highly recalcitrant plastic polymers. Moreover, while microbial strains belonging to the genera *Bacillus* and *Aspergillus* have been previously identified as candidates for breaking down polyethylene-based polymers as the sole carbon source (Nakei et al., 2022), this is the first report concerning the ability of *D. hansenii* and *C. denitrificans* species to degrade highly recalcitrant plastic polymers. While, expectations regarding the potential to degrade plastics by *C. denitrificans* REBF1 are high, as previous work published by Chawla and Singh Saharan (2014) has confirmed the ability of some strains to biodegrade triphenylmethane, a very recalcitrant dye introduced into the environment.

On the other hand, a consortium composed by *Pseudomonas aeruginosa* REBP5, *Pseudomonas alloputida* REBP7 and Non identified-Yeast RELP9 is proposed for the efficient degradation of LLDPE in powder format. The genera *Pseudomonas* has been commonly investigated in relation with plastic biodegradation, obtaining very promising results even in the most resistant plastic polymers like polyethylene (Hou et al., 2022). In this work, *P. alloputida* REBP7 and *P. aeruginosa* REBP5 were efficient biotechnological tools in the biodegradation of plastic waste, being even capable of forming consortia to promote improvements in the biodegradation process. Results obtained in this work are supported by others previously described by Roberts et al. (2020) and Muhonja et al. (2018), involving polyethylene degradation. Considering the enzymatic profile of *P. alloputida* REBP7, as well as the physical deterioration of the LLDPE detected by technology FESEM and the selective nature of the enrichment protocols used, the potential use of that strain in plastic degradation processes is evident. However, more caution should be exercised when using *P. aeruginosa* REBP5 as a member of a plastic-degrading consortium, taking into account that this species is commonly described as an opportunistic human pathogen (Qin et al., 2022), which would imply a more exhaustive control of its release into the environment.

The results derived from this work, although preliminary, are very promising in terms of the efficiency of biodegradation of LLDPE-contaminated matrices. However, any novel research work involves preliminary analyses, and results should be treated with some caution. In particular, the study involved a single plastic LLDPE, which is known to be highly recalcitrant. It should be noted that different plastics represent different substrates and, therefore, different microorganisms could compose other consortia with different plastic natures. However, the selection of LLDPE was made with careful deliberation, taking into account the fact that polyethylene is one of the most frequently used plastics in agriculture and industry. The extended usage of polyethylene increases the potential for environmental contamination. Consequently, finding a biologically and environmentally-friendly solution to this problem is of paramount importance. Though the results of this study are promising, the use of a single plastic may limit the generalizability of the findings. Future studies could explore the efficacy of the proposed biological solution on other types of plastics to determine its broader applicability. Additionally, the study was conducted under controlled laboratory conditions, and therefore, the efficacy of the proposed solution under real-world conditions requires further investigation.

## 5. Conclusion

The search for biotechnological techniques to solve the environmental pollution caused by the exponential increase in plastic waste could be a promising approach to mitigate this worrying problem. The strategy used in this work for the selection of natural consortia, including plastic contaminated microcosms followed by selective enrichment allowed to obtain stable consortia with capabilities to degrade the target polymer, LLDPE. In order to select and identify microorganisms capable of biodegrading recalcitrant plastic materials, a sequential and selective enrichment protocol from a microcosm artificially contaminated is proposed. This protocol is postulated as an excellent technique to obtain stable microbial consortia capable of growing at the expense of LLDPE in film or powder form. Additionally, results show that the plastic in powder form facilitates the microbial growth compared to its use in film format. Most strains from selective enrichment cultures showed a broad enzymatic profile related to plastic degradation, especially highlighting the Lipase (LIP), Cutinase1 (CUT1), Polyphenol oxidase (PO) activities, or the ability to degrade anthraquinone-type dyes *in vitro* in Remazol Brilliant Blue RBBR medium, which is closely related to ligninase activity. The strains *P. alloputida* REBP5 and *P. aeruginosa* REBP7 stood out because they were selected from prolonged enrichment cultures (PC) in the presence of LLDPE in powder form, and due to their metabolic versatility. For the same reason, although selected from prolonged enrichments in the presence of LLDPE in film format, the strains *Castellaniella denitrificans* REBF6 and *Debaryomyces hansenii* RELF10 stood out, although the enzymatic profiles were more restricted. It was also possible to conclude that some of the strains from the enrichment cultures in the presence of LLDPE, such as *Rhodococcus* REBF2, could collaborate in the prior degradation of additives accompanying the LLDPE polymer, facilitating the subsequent access of other microorganisms that are real degraders of the plastic structure. Thus, this study provides evidence that the enrichment of artificially contaminated microcosms is a promising approach for obtaining microbial consortia capable of efficiently degrading specific plastics, specifically LLDPE in this case. Furthermore, the members of these consortia displayed a degree of enzymatic complementarity, supporting the hypothesis that diverse microorganisms possess distinct capabilities, which allow for enhanced biodegradation of plastic materials.

## Data availability statement

The genomic sequences identified in this study have been deposited in the online repository National Center for Biotechnology Information (https://www.ncbi.nlm.nih.gov/) under accession numbers OQ411267, OQ411268, OQ411269, OQ411270 and OQ561300.

## Author contributions

ML, JS, and MS contributed to conception and design of the study as well as to the acquisition, analysis, and interpretation of data. VC, ME-G, AT, MJ, and JL-G contributed to experimental work. JS performed the statistical analysis. JS, MM-G, and FS-E wrote the first draft of the manuscript. All authors contributed to the manuscript revision, read, and approved the submitted version.

## Funding

This work has received funding from the Bio-based Industries Joint Undertaking (JU) under the European Union’s Horizon 2020 research and innovation programme under grant agreement no 887648. The JU receives support from the European Union’s Horizon 2020 research and innovation programme and the Bio-based Industries Consortium.

## Conflict of interest

The authors declare that the research was conducted in the absence of any commercial or financial relationships that could be construed as a potential conflict of interest.

## Publisher’s note

All claims expressed in this article are solely those of the authors and do not necessarily represent those of their affiliated organizations, or those of the publisher, the editors and the reviewers. Any product that may be evaluated in this article, or claim that may be made by its manufacturer, is not guaranteed or endorsed by the publisher.

## Footnotes

^1^ www.bioinformatics.org/sms/rev_comp.html
