# Supplementary figures and images for: Development of plastic-degrading microbial consortia by induced selection in microcosms
Source: Front Microbiol. 2023 Apr 11;14:1143769. doi: 10.3389/fmicb.2023.1143769 (PMC10126402; doi:10.3389/fmicb.2023.1143769)

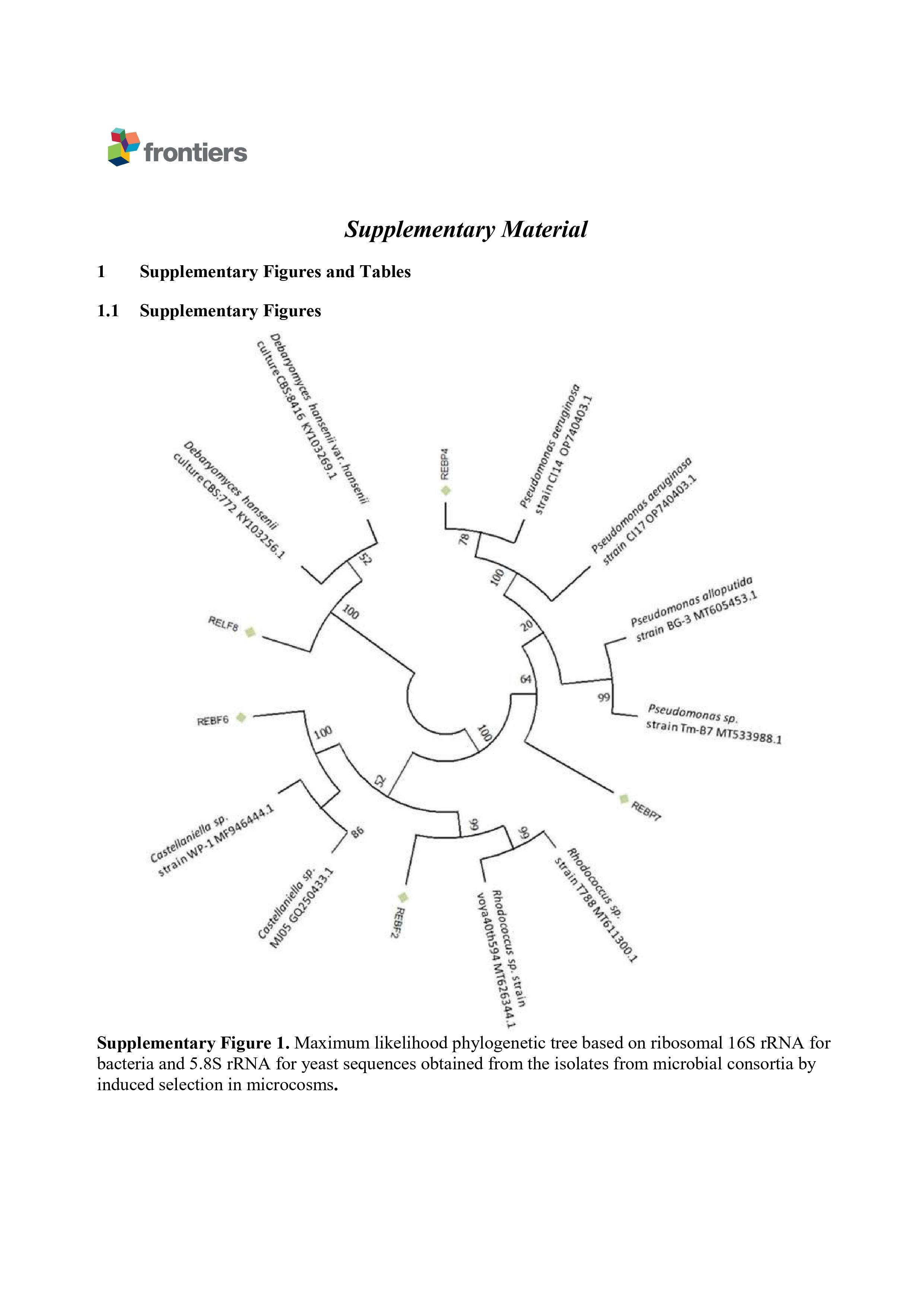

Supplement: Supplementary file 1 [file Image_1.JPEG]
